# Enhanced recovery after surgery for major orthopedic surgery: a narrative review

**DOI:** 10.1186/s43019-022-00137-3

**Published:** 2022-02-22

**Authors:** Yun Seong Choi, Tae Woo Kim, Moon Jong Chang, Seung-Baik Kang, Chong Bum Chang

**Affiliations:** 1 Department of Orthopedic Surgery, Seoul National University College of Medicine, Seoul, South Korea; 2 Department of Orthopedic Surgery, SMG-SNU Boramae Medical Center, Seoul, South Korea; 3 Department of Orthopedic Surgery, Seoul National University Bundang Hospital, 82, Gumi-ro 173 Beon-gil, Bundang-gu, Seongnam-si, Gyeonggi-do 13620 South Korea

**Keywords:** Enhanced recovery after surgery, Orthopedic surgery, Components, Outcomes, Implementation

## Abstract

**Background:**

With increasing interest in enhanced recovery after surgery (ERAS), the literature on ERAS in orthopedic surgery is also rapidly accumulating. This review article aims to (1) summarize the components of the ERAS protocol applied to orthopedic surgery, (2) evaluate the outcomes of ERAS in orthopedic surgery, and (3) suggest practical strategies to implement the ERAS protocol successfully.

**Main body:**

Overall, 17 components constituting the highly recommended ERAS protocol in orthopedic surgery were identified. In the preadmission period, preadmission counseling and the optimization of medical conditions were identified. In the preoperative period, avoidance of prolonged fasting, multimodal analgesia, and prevention of postoperative nausea and vomiting were identified. During the intraoperative period, anesthetic protocols, prevention of hypothermia, and fluid management, urinary catheterization, antimicrobial prophylaxis, blood conservation, local infiltration analgesia and local nerve block, and surgical factors were identified. In the postoperative period, early oral nutrition, thromboembolism prophylaxis, early mobilization, and discharge planning were identified. ERAS in orthopedic surgery reduced postoperative complications, hospital stay, and cost, and improved the patient outcomes and satisfaction with accelerated recovery. For successful implementation of the ERAS protocol, various strategies including the standardization of care system, multidisciplinary communication and collaboration, ERAS education, and continuous audit system are necessary.

**Conclusion:**

The ERAS pathway enhanced patient recovery with a shortened length of stay, reduced postoperative complications, and improved patient outcomes and satisfaction. However, despite the significant progress in ERAS implementation in recent years, it has mainly focused on major surgeries such as arthroplasty. Therefore, further efforts to apply, audit, and optimize ERAS in various orthopedic surgeries are necessary.

## Background

Enhanced recovery after surgery (ERAS) is a multimodal, multidisciplinary, and evidence-based protocol that promotes fast recovery by reducing the patient’s surgical stress, organ dysfunction, and optimizing their physiologic function [1]. ERAS was first introduced in colorectal surgery by Kehlet, a Danish surgeon in 1997, and ERAS has spread to other surgical specialties, showing significant improvements in the clinical outcomes and costs [2].

With great interest in the ERAS pathway, literature on ERAS in orthopedic surgery is also rapidly increasing [3–6]. Recently, the ERAS Society reported the ERAS guidelines for hip–knee arthroplasty and spine surgery [4, 5], and even now, additional evidence supporting each ERAS component is being added. Numerous studies have also investigated the ERAS outcomes in orthopedic surgery from various aspects, including the length of stay, complications, clinical outcomes, and medical costs, and have reported promising results.

Nevertheless, the implementation of the ERAS protocol in orthopedic practice remains a difficult issue [7]. Recently, a number of studies on ERAS have reported various obstacles in ERAS implementation, such as reduced compliance and ERAS protocol deviation by providers and patients [8, 9]. To transfer the ERAS protocol to be practiced successfully, practical strategies based on a sufficient understanding of ERAS components and their outcomes are necessary.

Therefore, this review article aims to (1) summarize the components of the ERAS protocol applied to orthopedic surgery, (2) evaluate the outcomes of ERAS in orthopedic surgery, and (3) suggest practical strategies to implement the ERAS protocol successfully.

## Components of ERAS protocol

The ERAS components cover all perioperative periods, including preadmission, preoperative, intraoperative, and postoperative periods. In each period, multimodal treatments are given by different professional departments, such as surgery, anesthesia, and nursing (Table 1)**.**

**Table 1:** Summary of the ERAS components for orthopedic surgery

Period	Component	Contents
Preadmission	Preadmission counseling	Patients should be informed of the treatment they receive, what to expect, and their role in the recovery process during their hospital stay
	Optimization of medical condition	Underlying disease: underlying disease should be identified through blood test, imaging tests, and history taking, and optimized with the help of a specialist Smoking: it is recommended to stop smoking at least 4 weeks before total joint arthroplasty Alcohol: alcohol cessation is recommended before total joint arthroplasty Malnutrition and anemia: preoperative correction of malnutrition and anemia is recommended before total joint arthroplasty
Preoperative	Avoid prolonged fasting	Clear fluid was allowed 2 h before induction of anesthesia, and solid food was allowed 6 h before, but routine intake of carbohydrates before surgery is still not recommended
	Multimodal analgesia	NSAID, paracetamol: decrease postoperative pain and reduce supplemental analgesic (opioid) use following hip and knee replacement Gabapentinoid: routine use is not recommended because of insufficient evidence Antidepressant (duloxetine): significantly reduce opioid use and nausea Opioid: current trend is to implement multimodal analgesia without opioid Corticosteroid: can be used as a drug for preemptive analgesia with NSAID and pregabalin
	PONV	Corticosteroids (dexamethasone), serotonin (5HT3) antagonists (ondansetron), and dopamine (D2) antagonists (droperidol) are commonly used to prevent PONV
Intraoperative	Anesthetic protocol	Anesthesia techniques (neuraxial versus general) more suitable for orthopedic surgery have not yet been clarified. Routine use of spinal opioids or epidural anesthesia is unreasonable
	Prevent hypothermia	Normal body temperature should be maintained intraoperatively through prewarming and humidification of anesthetic gases, warming IV and irrigation fluids, and warming blankets
	Fluid management	Fluid management should be adjusted to maintain the normal state of the body fluid compartment, facilitate the excretion of waste, and return to oral intake as early as possible after surgery
	Urinary catheterization	should be removed as soon as possible, ideally within 24 h after completion of surgery. However, it should not be used routinely, and should be determined by patients’ condition
	Antimicrobial prophylaxis	Antibiotics prophylaxis and dilute betadine lavage can prevent surgical site infection and periprosthetic joint infection, but preoperative hair removal is not recommended
	Blood conservation	Tranexamic acid is effective in reducing blood loss and transfusion rate in orthopedic surgery
	LIA ad local nerve block	LIA is effective for TKA and is more suitable for the ERAS protocol than a nerve block, which can inhibit early mobilization by blocking the motor nerve
	Surgical factors	Surgical approach: there is no conclusive evidence that choice of surgical approach accelerates the achievement of discharge criteria Tourniquet, drainage: routine use is not recommended in orthopedic surgery ICE therapy: effective in relieving pain, reducing swelling, and improving ROM
Postoperative period	Early oral nutrition	An early return to normal diet as soon as patients feel able is recommended
	Thromboembolism prophylaxis	Patients should be mobilized as soon as possible after surgery and should receive appropriate antithrombotic prophylactic treatment
	Early mobilization	Patients should be mobilized as early as they are able because prolonged bed rest causes thromboembolism, pulmonary complications, and muscle atrophy
	Discharge planning	Objective discharge criteria should be established so that patients can be discharged directly to their home

*NSAID* nonsteroidal anti-inflammatory drugs; *PONV* prevention of postoperative nausea and vomiting; *IV* intravenous; *LIA* local infiltration analgesia; *TKA* total knee arthroplasty; *ERAS* enhanced recovery after surgery; *ROM* range of motion

### Preadmission counseling

Preoperative education is the process of explaining to a patient their treatment and recovery process during hospitalization. Although previous studies have reported that preoperative education does not reduce postoperative complications, improve pain, or shorten the hospital stay [10], good preoperative education can significantly reduce patient anxiety and emotional stress while increasing patient satisfaction and increasing patient trust [10, 11]. Kaye et al. reported on the importance of informing patients that postoperative pain might occur even if painkillers are used and to control the patients’ unrealistic expectations for postoperative results[12].

### Optimization of medical condition

Before surgery, it is necessary to check the patient's general health and optimize it to a condition suitable for surgery. A checklist to consider before surgery includes underlying disease (hypertension, diabetes mellitus, respiratory diseases, heart failure, chronic kidney disease, etc.), smoking and alcohol history, and nutritional status. The patient's underlying disease is identified through blood tests, imaging tests, and history-taking, and is usually optimized with the help of a specialist. It is also recommended to quit smoking. Pierre et al. reported that the group who quit smoking for more than 4 weeks had a 25% reduction in respiratory complications compared with the control group [13]. It is also recommended to abstain from alcohol. Eliasen et al. reported that alcohol consumption is associated with infection, lung complications, and long-term hospital stays [14]. It is also necessary to check the nutritional condition before surgery. Gu et al. reported that the albumin level is < 3.5 dg/L and there is a greater possibility of postoperative wound complications [15]. Preoperative anemia was associated with high postoperative transfusion rates and was significantly associated with postoperative infections, poorer physical function, and increased hospital stay and mortality [16–19]. In particular, among patients undergoing total hip arthroplasty (THA) and total knee arthroplasty (TKA), preoperative anemia was very high, ranging from 24 ± 9% to 44 ± 9% [20], and more than 42% of hip fracture surgery patients present with anemia on admission [21, 22]. Preoperative anemia correction reduced the need for postoperative blood transfusion and could contribute to improving the patient’s outcome [18, 23–25].

### Avoid prolonged fasting

Previously, most patients had implemented nothing per oral after midnight (NPO) before surgery; however, since recently, clear fluid is allowed 2 h before the induction of anesthesia, and solid foods are allowed 6 h prior to the induction [26]. Prolonged fasting can cause catabolic conditions, which can increase the stress response to surgery and cause insulin resistance and hyperglycemia, prolonging the recovery period. In contrast, carbohydrate loading has been shown to decrease the insulin resistance and infection and increase the patient’s well-being [27]. Carbohydrate loading involves taking the patients into surgery in a “metabolically fed state,” which results in less protein and muscle loss after surgery [28]. However, two randomized controlled trials (RCTs) have compared the effects of carbohydrate loading versus preoperative fasting on glucose control in the spinal surgery population; neither could prove the advantage of carbohydrate loading [29, 30]. Therefore, the routine use of carbohydrates before surgery is still not recommended.

### Multimodal analgesia

The concept of multimodal analgesia is to combine various drugs and multiple routes of administration to achieve pain control, and it was reported to be effective for pain control in orthopedic surgery. Lee et al. stated that multimodal analgesia was very efficient in reducing postoperative pain in upper extremity surgery, such as for distal humerus fracture, elbow fracture and dislocation, multiple elbow ligament injuries, and osteoarthritis [31]. A combination of drugs with different mechanisms simultaneously targets different points in the pain pathway [32]. Nonsteroidal anti-inflammatory drugs (NSAID)s have a great advantage in reducing the pain after arthroplasty [33]. However, they has side effects, including gastrointestinal (GI) tract ulcers, renal dysfunction, and cardiovascular disease (CVD). Paracetamol is effective in reducing mild to moderate pain, and side effects, such as nausea and vomiting (N/V), skin irritation, and thrombocytopenia, are less common [34]. Gabapentinoids inhibit the release of neurotransmitters, such as substance P, resulting in decreased nerve excitability and pain. However, due to insufficient evidence to demonstrate the effectiveness of gabapentinoids, the routine use of gabapentinoids in the ERAS protocol is not recommended [5, 35]. Opioids have numerous side effects, including drowsiness, respiratory depression, N/V, and urinary retention; therefore, the current trend is to implement multimodal analgesia without opioids [5]. In addition, antidepressants (duloxetine) are effective in reducing opioid use and nausea [36]. Corticosteroids are potent anti-inflammatory agents that have been widely used in various perioperative settings to reduce the postoperative pain. In particular, previous studies have reported that corticosteroids can be used as a drug for preemptive analgesia with NSAIDs and pregabalin to control post-TKA pain and effectively control postoperative pain without wound complications [37, 38].

### Prevention of postoperative nausea and vomiting (PONV)

PONV is one of the most distressing symptoms in patients, along with postoperative pain. The well-known risk factors for PONV include the following: (1) female sex, (2) non-smoking status, (3) prior history of motion sickness, and (4) use of postoperative opioids [39]. The treatment for PONV is mainly managed by pharmacological treatment. Popular drugs include corticosteroids (dexamethasone), serotonin-5-HT3 antagonists (ondansetron), and dopamine (D2) antagonists (droperidol), which have been shown to reduce PONV more effectively [40]. Koh et al. reported that PONV could be significantly reduced when dexamethasone was used together with 5HT3 antagonists before TKA compared with when 5HT3 antagonists were used alone [37].

### Anesthetic protocol

In orthopedic lower limb surgery, neuraxial anesthesia is generally used more frequently than general anesthesia [41]. This is because neuraxial anesthesia reduces pulmonary complications, PONV, postoperative pain, and ileus, thus reducing the hospital stay [42]. However, previous studies have reported no significant difference in the mortality, surgical duration, surgical site infections (SSI), nerve palsy, thromboembolic disease, and blood loss between neuraxial anesthesia and general anesthesia [43]; moreover, urinary bladder dysfunction is more reduced under general anesthesia [5]. Additional studies on the two anesthesia techniques are needed. In spinal anesthesia, opioids seem to reduce the pain after surgery [44]; however, they are not easy to use because side effects, such as delay of recovery, urinary retention, response, and PONV, have been identified [45]. In epidural analgesia, which is performed for postoperative pain control, the advantage of reducing pain seems obvious; however, it has side effects, including delayed recovery, hypotension, and urinary retention [46]. Therefore, the routine use in THA and TKA seems unreasonable.

In spine surgery, there was no difference between nongeneral and general anesthesia in terms of readmission rates, complications, and length of stay (LOS) [47]. However, many options exist for general anesthesia due to the wide range of available drugs and delivery methods. Brown and Gille et al. reported that the use of neuromuscular blockade reduced airway pressure and muscle damage associated with prolonged retraction during spine surgery [48, 49].

### Prevent hypothermia

Hypothermia is common in patients who have undergone orthopedic surgery [50]. Previous studies have reported the incidence of hypothermia for THA, TKA, and hip fracture to be 26.3%, 28.0%, and 10%, respectively [51, 52]. In addition, Williams et al. reported that inadvertent hypothermia was significantly associated with a higher 30-day readmission rate and a 30-day mortality in elderly patients with hip fracture [52]. If the patient fails to maintain normothermia during surgery and heat loss occurs, stress responses, such as nitrogen loss and the release of cortisol and catecholamine, increase [1]. This may increase the infection, coagulopathy, blood transfusion rate, cardiovascular complications, and opioid need, which may adversely affect the postoperative outcome [53]. Lower preoperative temperature, female sex, lower body mass index, older age, adult reconstruction by specialty, and hip and pelvic procedures by anatomic region are known risk factors for hypothermia during orthopedic surgery [50]. Methods of maintaining normothermia include prewarming and humidification of anesthesia gas, preheating of intravenous (IV) and irrigation fluids, and forced-air warming of blankets and devices [5]. Rosenkilde et al. reported that unintended perioperative hypothermia in total joint arthroplasty was identified in 13% of the patients in the prewarming group and 43% of the patients in the control group [54].

### Fluid management

There are three fluid therapies: (1) liberal fluid therapy, (2) restricted fluid therapy, and (3) goal-directed fluid therapy (GDT) [55]. Liberal fluid therapy involves injecting a large amount of fluid to improve the tissue oxygen delivery and maintain the urine output because the patients undergoing surgery are in a hypovolemic state with long fasting times and high blood loss [56]. However, this therapy can cause hypervolemia, which can lead to tissue edema, pulmonary edema, and cardiac complications [57]. Therefore, new management methods, such as restricted fluid therapy, have been introduced to prevent such occurrences. In restrictive fluid therapy, fluid is injected to achieve zero balance [58]. Although this may avoid hypervolemia, it can cause hypotension and decrease perfusion to the vital organs, causing organ dysfunction, such as acute kidney injury [57]. GDT monitors the hematologic parameters (pulse pressure variation, delta stroke volume, central venous pressure, and urine output) before and after surgery to achieve normal use of fluid or inotropes [59]. GDT is highly recommended in the ERAS protocol. However, unlike colorectal surgery, where GDT is important, it does not seem to be as important for surgeries, such as THA and TKA. This is because the loss of blood and fluid is lower than expected in arthroplasty; additionally, oral intake is more likely to occur sooner after arthroplasty. Fluid management should be adjusted to maintain the normal state of the body fluid compartment, facilitate the excretion of waste, and return to oral intake as early as possible [5].

### Urinary catheterization

Postoperative urinary retention (POUR) can lead to urinary tract infection (UTI), wound infection, and periprosthetic infection [60]. Previous studies have demonstrated that indwelling urinary catheterization prevents POUR and significantly reduces the incidence of UTI [61]. Therefore, several orthopedic surgeons perform urinary catheterization in these patients. However, recent studies have reported that POUR does not increase without indwelling urinary catheterization owing to the development of surgical and anesthesia techniques [62], and the recent trend toward rapid recovery and early mobilization of patients has led to the direction of not performing indwelling urinary catheterization [63]. However, when the operation time is long and it is necessary to check the urinary output, indwelling urinary catheterization should be used as a guide for fluid resuscitation. When indwelling urinary catheterization is used, it should be removed as soon as possible, ideally within 24 h after the completion of surgery [5]. Indwelling urinary catheterization should not be used routinely, and it should be determined based on the patient’s condition.

### Antimicrobial prophylaxis

Antibiotic prophylaxis is a very important management strategy to prevent SSI and periprosthetic joint infection (PJI) in patients undergoing arthroplasty [64]. Several orthopedic surgeons refer to the Surgical Care Improvement Project (SCIP) guidelines for antibiotic prophylaxis.

According to the SCIP guideline [65],

- (1) In principle, prophylactic antibiotics should be injected within 1 h prior to surgical incision, but if the patient needs to be injected with vancomycin or fluoroquinolone, it should be injected within 2 h before surgical incision.
- (2) Patients should be prescribed a suitable antibiotic depending on the type of surgery.
- (3) Preventive antibiotics should be stopped within 24 h of the completion of the surgery.

Previous studies have reported that patients who received antibiotics more than 2 h before incision had an approximately two- to sixfold increased risk of SSI [66]. In addition, if methicillin-resistant *Staphylococcus aureus* and coagulase-negative staphylococci are identified, vancomycin should be administered, and if the patient is allergic to penicillin and cephalosporin, fluoroquinolone should be administered. Patients undergoing orthopedic surgery are most likely to be infected with *S. aureus* and streptococcal species. Therefore, first-generation cephalosporins (typically cefazolin) are commonly administered [67]. Moreover, it is recommended that prophylactic antibiotics should be terminated within 24 h even if there is drainage after surgery [68]. In addition, it was reported that dilute betadine lavage before surgical wound closure in total joint arthroplasty is an effective measure to reduce the risk of acute postoperative PJI [69], and that skin preparation using either alcohol-based iodine or chlorhexidine solution could provide a more favorable longer-lasting effect for skin antisepsis in spine surgery [4]. Meanwhile, there was consensus that hair removal before surgery has no significant effect on reducing the incidence of SSI; however, shaving increases the incidence of SSI [70].

### Blood conservation

Significant blood loss can occur in tumor surgery, pelvic surgery, and primary and revision arthroplasty. Blood loss has been treated by blood transfusion; however, this is not an easy option because it can cause serious complications, such as infection and disease transmission [71]. A decrease in the blood loss and transfusion rate may be achieved using combined local and systemic tranexamic acid [5]. Tranexamic acid is an antifibrinolytic drug that competitively suppresses the plasmin activation of plasminogen, thereby preventing the destruction of fibrin clotting and reducing blood loss. Gautam et al. revealed that tranexamic acid was effective in reducing blood loss and blood transfusion in patients undergoing TKA [72], and Haj-Younes et al. also reported that intravenous tranexamic acid reduced the proportion of patients requiring blood transfusions when undergoing hip fracture surgery [73]. In addition, tranexamic acid can be administered via topical routes in addition to the intravenous and oral routes. Indeed, Wong et al. added tranexamic acid to normal saline at the end of surgery and applied it topically to the joint surface, demonstrating its effectiveness in reducing the blood loss [74]. However, it is important to be aware that it can increase the risk of deep vein thrombosis (DVT) [75].

### Local infiltration analgesia (LIA) and local nerve block

LIA is the administration of painkillers around the surgical site during surgery, mostly in TKA. LIA is mainly composed of a combination of multimodal analgesics, including local anesthetics, NSAIDs, opioids, and steroids, and is effective for pain that can occur 6–12 h after surgery [76]. In fact, it is a technique used by many orthopedic surgeons for patients undergoing TKA. In addition to TKA, it was reported that LIA was also effective in ankle and shoulder surgery. Li et al. stated that LIA with ropivacaine could provide better early postoperative pain management, with a reduction in VAS scores for ankle fracture surgery [77]. Moreover, Sicard et al. reported that LIA was not less effective than interscalene nerve block for early postoperative pain control after total shoulder arthroplasty [78]. However, in THA, it was reported that LIA did not significantly reduce the postoperative pain [79]. Nerve block is a technique used for analgesic purposes, especially femoral nerve block, which has been proven to significantly reduce postoperative pain compared with other epidural analgesia or patient-controlled analgesia (PCA) [80]. However, the sciatic nerve block did not offer any particular advantage compared with other analgesic techniques for hip or knee replacement [80]. Since nerve block can block the motor nerve, LIA is generally considered to be a safer technique. The motor block can be harmful to muscle function and makes early mobilization difficult [81]; therefore, the routine use of nerve block techniques is not recommended in the ERAS protocol.

### Surgical factors

There is insufficient evidence that one particular surgical approach may shorten the length of the patient's hospitalization period compared with other surgical approaches in arthroplasty and spinal surgery [4, 82]. The use of tourniquets when performing TKA can generally improve vision by reducing bleeding during surgery [83]. However, the routine use of tourniquets in the ERAS protocol is not recommended, as DVT, peripheral nerve damage, and skin irritation have also been reported. Additionally, hematoma, which may occur after arthroplasty, acts as a good medium for bacteria causing SSI or PJI; therefore, several orthopedic surgeons usually connect the drain to the surgical site and complete the surgery. However, there have been reports that blood loss and transfusion rate can be increased and retro-infection can occur through a drain in THA [84], and four RCTs showed that drains did not reduce the incidence of either SSI or postoperative hematoma during spinal surgery [85–88]. Therefore, the routine use of drains is not recommended in the recent ERAS protocol. Meanwhile, nonpharmaceutical treatments, such as cryotherapy, play a role in addressing immediate postoperative complications, such as pain and swelling. Zhong et al. reported that applying an ice pack after TKA is effective in relieving pain, reducing swelling, and improving the range of motion (ROM) [89].

### Early oral nutrition

No studies have investigated the direct relationship between postoperative nutritional supplementation and accelerating the achievement of discharge criteria. However, encouraging patients to eat and drink as soon as possible is considered an essential component of the ERAS protocol, as returning to normal food intake can help patients return to normal behavior [5].

### Thromboembolism prophylaxis

Venous thromboembolism (VTE) has been reported to occur in approximately 40–60% of major orthopedic surgeries (THA, TKA, and hip fracture) without the use of preventive anticoagulants [90, 91]. The drugs commonly used for VTE prophylaxis include low-molecular-weight heparin, warfarin, rivaroxaban, fondaparinux, and aspirin [92]. Mechanical therapy commonly used for VTE prophylaxis includes graduated compression stockings and intermittent pneumatic compression devices (IPCDs) [92]. The main VTE prophylaxis guidelines are the American College of Chest Physicians (ACCP) guidelines and the American Academy of Orthopedic Surgeons (AAOS) guidelines. The recently announced 10th Amendment ACCP guidelines consider major orthopedic surgeries such as THA, TKA, and hip fracture a high-risk group for VTE and recommend that one of the drugs described above be used for at least 10–14 days and, if possible, up to 35 days after surgery [91]. However, although VTE prophylaxis reduces the frequency of thrombosis, it seems that the proportion of asymptomatic thrombosis is large, and the evidence that such thrombosis progresses to symptomatic thrombosis or pulmonary embolism is weak [93]. Therefore, AAOS considers the side effects of antithrombotic agents, such as bleeding, and suggests a guideline to prevent them, with additional emphasis on symptomatic DVT and pulmonary embolism (PE) [1, 53]. The guidelines that recommended a period of 10–35 days for VTE prophylaxis after THA, TKA, and hip fracture did not consider “early mobility.” Therefore, the AAOS guidelines considered “early mobility.” In fact, early mobility can reduce the occurrence of DVT, thereby reducing the period of prophylaxis and hospitalization [94].

### Early mobilization

The patient should be mobilized as soon as possible after surgery. If the patient prolongs bed rest, the patient will have thromboembolism, pulmonary complications, and muscle atrophy [95] and eventually the hospitalization period will be extended. In fact, Guerra et al. reported that early mobility reduced hospitalization by an average of 1.8 days in total joint arthroplasty [96], and Deng et al. reported that patient‐reported outcome measures (PROMs) score of the early mobilization group was significantly better than that of the late mobilization group after distal radius fracture surgery [97].

### Discharge planning

The ERAS of THA and TKA may include the objective discharge criteria for patients to be discharged directly to their home. It is necessary to clearly define the degree of independent dressing, the ability to get in and out of bed, the ability to sit and get up in a chair/toilet, the ability to be independent of personal care, and the ability to walk using a walker or crutches [98].

## Outcomes of ERAS in orthopedic surgery (Table 2)

**Table 2:** Summary of the outcomes of ERAS in orthopedic surgery

Study	Year	Surgery type	Study type	Study/control (N)	Result
Auyong et al. [100]	2015	TKA	Retrospective	126/126	Reduced LOS, transfusion rate, postoperative nausea
Zhu et al. [11]	2017	THA, TKA	Meta-analysis	4205/5731	Reduced LOS, complication
Gwynne et al. [99]	2017	TKA	Prospective	528/507	Reduced LOS, Oxford knee score was improved
Deng et al. [101]	2018	THA, TKA	Meta-analysis	6944/9755	Reduced LOS, mortality, transfusion, complications
Hu et al. [112]	2019	Joint, fx, spine surgery	Meta-analysis	9700/11,143	Reduced incidence of postoperative complications, 30-day mortality rate
Garriga et al. [102]	2019	TKA	Retrospective	486,579	Reduced LOS, bed-day costs, complications Oxford knee score was improved
Kang et al. [109]	2019	Intertrochanteric fx	Prospective	50/50	Reduced LOS, complications, readmission rate, opioid consumption Harris hip score was improved
Jiang et al. [103]	2019	TKA	Prospective	106/141	Reduced postoperative pain, LOS, blood loss Knee society score and ROM degree were improved
Xiao et al. [113]	2019	Close reduction of distal radius fx	Prospective	72/114	Reduced complications, improved patient-rated wrist evaluation score
Yin et al. [104]	2020	Intertrochanteric fx	RCT	30/30	Reduced LOS
Wang et al. [105]	2020	Spine surgery	Retrospective	96/96	Reduced LOS
Ripollés-Melchor et al. [106]	2020	THA, TKA	Prospective	163/517	Reduced LOS, complications within 30 days after surgery
Pritchard et a1 [115]	2020	THA, TKA	Systematic review	–	Improved cost-effectiveness
Tong et al. [110]	2020	Spine surgery	Systematic review	–	Reduced LOS, opioid consumption, improved cost-effectiveness
Frassanito et al. [107]	2020	THA, TKA	Prospective	207	Reduced LOS, opioid consumption, improved satisfaction score
Sun et al. [111]	2020	ACL reconstruction	Prospective	30/30	Reduced LOS Improved satisfaction score, Lysholm knee scoring scale
Leiss et al. [114]	2021	THA	Retrospective	109	Harris hip score, WOMAC score, and EQ-5D were improved Improved satisfaction score
Liu et al. [108]	2021	Hip fx	Meta-analysis	9869	Reduced LOS, TTS, and complications
Morrell et al. [3]	2021	THA, TKA	Systematic review	2428/5361	Reduced LOS

*N* number; *THA* total hip arthroplasty; *TKA* total knee arthroplasty; *LOS* length of stay; *fx* fracture; *ROM* range of motion; *RCT* randomized controlled trial; *TTS* time to surgery; *WOMAC* Western Ontario and McMaster Universities Osteoarthritis Index

### LOS

ERAS reduced LOS [3, 11, 99–111]. In particular, Morrell et al. reported that 2428 patients in the ERAS group had a significant decrease in the LOS after THA and TKA compared with 5361 patients in the control group [3]. Furthermore, Sun et al. stated that, after anterior cruciate ligament reconstruction, 30 patients in the ERAS group had a significant decrease in LOS compared with 30 others in the control group (7.3 days versus 11.5 days, respectively) [111]. Wang et al. reported that the ERAS group had a shorter median LOS than the non-ERAS group in short-level lumbar fusion (12.30 ± 3.03 of ERAS group; 15.50 ± 1.88 in non-ERAS group) [105].

### Complication

There were fewer incidences of complications in the ERAS group than in the non-ERAS group [11, 101, 102, 106, 108, 109, 112, 113]. Zhu et al. reported that 3262 patients in the ERAS group had a significant decrease in complications after THA and TKA compared with 4527 patients in the control group [11]. Xiao et al. reported that 72 patients in the ERAS group had a significant decrease in close reduction of distal radius fracture compared with 114 patients in the control group [113]. In addition, Liu et al. also described that 1852 patients in the ERAS group had a significant decrease in complications after hip fracture surgery compared with 1302 patients in the control group [108].

### PROM

Patients in the ERAS group had better postoperative PROM scores than those in the non-ERAS group [99, 102, 103, 107, 109, 111, 113, 114]. In particular, Kang et al. reported that patients in the ERAS group had better 3-month Harris hip scores after intertrochanteric fracture surgery than those in the non-ERAS group [109], and Frassanito et al. reported that satisfaction scores of patients who underwent total joint arthroplasty with the ERAS protocol improved [107]. Leiss et al. conducted a retrospective study of 109 patients who underwent cementless THA with the ERAS protocol; their Harris hip score, Western Ontario and McMaster Osteoarthritis Index (WOMAC) score, EuroQol-5 Dimensions (EQ-5D), and patient satisfaction showed continuous improvement over a follow-up of 12 months after surgery [114].

### Cost

The ERAS protocol has shown significant cost-effectiveness while reducing the LOS and complications [102, 110, 115]. Garriga et al. evaluated 486,579 patients who underwent TKA and reported that LOS and bed-day costs decreased from 5.8 to 3.7 days and from £7607 to £5276, from April 2008 to December 2016 [102]. Tong et al. reviewed 22 studies evaluating the cost-effectiveness of adult spine surgery with the ERAS protocol and reported that ERAS may improve cost-effectiveness to varying degrees for spinal procedures [110].

## Implementation of ERAS: from protocol to practice

Although well-established ERAS protocols have shown promising outcomes, their implementation in practice remains a difficult issue. An increasing number of studies on ERAS have reported various obstacles to ERAS implementation, such as reduced compliance and ERAS protocol deviation by providers and patients [116–120]. Several practical strategies are required for the successful implementation of the ERAS protocol.

### Multimodal and standardized care system

Each ERAS element is closely related and has a synergistic effect with the others [6]. For example, early mobilization can be achieved by pain control, reduced bleeding, improved PONV, and restricted fluid therapy. Therefore, to implement the ERAS protocol effectively, a multimodal approach to utilize all available ERAS elements is essential, and the standardization of patient care systems, such as set order or clinical pathway, is necessary [6, 117]. Foni et al. described that the implementation of CP in TKA was related to a reduction in the length of hospital stay from 6.3 to 4.9 days, and a reduction in cost compared with the control group [121]. Schwarzkopf et al. also reported that the postoperative PROM score was significantly better than the preoperative PROM score when CP was implemented [122].

### Multidisciplinary communication and collaboration

ERAS elements are carried out by various professional departments, such as surgery, anesthesia, nursing, and physical therapy. The sharing of patients’ information and communication between ERAS participants are key to successful multidisciplinary collaboration [119]. Formal structures to encourage communication, such as consistent ERAS team meetings, can be helpful for multidisciplinary collaboration [116].

### ERAS education

Traditional typical care, general resistance to change, and individual surgeon preference are known to be obstacles to ERAS adherence [116, 117]. To reduce the provider-derived pathway deviation unaccompanied by clinical rationale, ERAS education for all providers is necessary [118]. The empowerment of patients by ERAS education can also promote them to work toward recovery [119].

### Continuous audit system

To improve ERAS compliance, continuous feedback with an audit system is necessary. Ament et al.[123] reported that the loss of audit feedback can be a reason for the diminished effectiveness of the ERAS pathway. Multidisciplinary communication and collaboration can work based on the audit system, and the audit system can also update the ERAS protocol in light of new evidence [6].

## Future of ERAS

Evidence-based ERAS protocols in orthopedic surgery have been well established with numerous supporting studies, especially in hip and knee arthroplasty and spinal surgery. ERAS in orthopedic surgery successfully enhanced patient recovery with shortened LOS, reduced postoperative complications, and improved clinical outcomes and satisfaction. However, ERAS studies on other types of surgery, including fracture, arthroscopy, and trauma, are very limited, and which elements are more important among numerous ERAS items still remains unclear. For the successful implementation of ERAS in various fields of orthopedic surgery, future studies need to focus on the optimization of the ERAS protocol depending on each surgery type through continuous audit and feedback.

## Conclusion

The ERAS pathway enhanced patient recovery with a shortened LOS, reduced postoperative complications, and improved patient outcomes and satisfaction. However, despite the significant progress in ERAS implementation in recent years, it has mainly focused on major surgeries such as arthroplasty. Therefore, further efforts to apply, audit, and optimize ERAS in various orthopedic surgeries are necessary.

## Data Availability

Not applicable.

## Abbreviations

ERAS: Enhanced recovery after surgery
THA: Total hip arthroplasty
TKA: Total knee arthroplasty
NPO: Nothing per oral after midnight
NSAID: Nonsteroidal anti-inflammatory drugs
GI: Gastrointestinal
CVD: Cardiovascular disease
N/V: Nausea and vomiting
PONV: Postoperative nausea and vomiting
SSI: Surgical site infections
IV: Intravenous
GDT: Goal-directed fluid therapy
POUR: Postoperative urinary retention
UTI: Urinary tract infection
PJI: Periprosthetic joint infection
SCIP: Surgical Care Improvement Project
DVT: Deep vein thrombosis
LIA: Local infiltration analgesia
PCA: Patient-controlled analgesia
ROM: Range of motion
VTE: Venous thromboembolism
IPCD: Intermittent pneumatic compression devices
ACCP: American College of Chest Physicians
AAOS: American Academy of Orthopedic Surgeons
LOS: Length of stay
PROM: Patient‐reported outcome measures
WOMAC: Western Ontario and McMaster Osteoarthritis Index
EQ-5D: EuroQol-5 Dimensions
